# Cobalt containing glass fibres and their synergistic effect on the HIF-1 pathway for wound healing applications

**DOI:** 10.3389/fbioe.2023.1125060

**Published:** 2023-03-10

**Authors:** Anu K. Solanki, Hélène Autefage, Antonio R. Rodriguez, Shweta Agarwal, Joaquin Penide, Muzamir Mahat, Thomas Whittaker, Amy Nommeots-Nomm, Elena Littmann, David J. Payne, Anthony D. Metcalfe, Felix Quintero, Juan Pou, Molly M. Stevens, Julian R. Jones

**Affiliations:** ^1^ Department of Materials, Imperial College London, London, United Kingdom; ^2^ Department of Bioengineering, Imperial College London, London, United Kingdom; ^3^ Institute of Biomedical Engineering, Imperial College London, London, United Kingdom; ^4^ Dpto. Fisica Aplicada, Universidad de Vigo, E.I. Industrial, Vigo, Spain; ^5^ Faculty of Applied Sciences, Universiti Teknologi MARA, Shah Alam, Malaysia; ^6^ Research Complex at Harwell, Harwell Science and Innovation Campus, Didcot, United Kingdom; ^7^ Healthcare Technologies Institute, School of Chemical Engineering, University of Birmingham, Birmingham, United Kingdom

**Keywords:** bioactive glass, angiogenesis, laser spinning, wound healing, hypoxia

## Abstract

**Introduction and Methods:** Chronic wounds are a major healthcare problem, but their healing may be improved by developing biomaterials which can stimulate angiogenesis, e.g. by activating the Hypoxia Inducible Factor (HIF) pathway. Here, novel glass fibres were produced by laser spinning. The hypothesis was that silicate glass fibres that deliver cobalt ions will activate the HIF pathway and promote the expression of angiogenic genes. The glass composition was designed to biodegrade and release ions, but not form a hydroxyapatite layer in body fluid.

**Results and Discussion:** Dissolution studies demonstrated that hydroxyapatite did not form. When keratinocyte cells were exposed to conditioned media from the cobalt-containing glass fibres, significantly higher amounts of HIF-1α and Vascular Endothelial Growth Factor (VEGF) were measured compared to when the cells were exposed to media with equivalent amounts of cobalt chloride. This was attributed to a synergistic effect of the combination of cobalt and other therapeutic ions released from the glass. The effect was also much greater than the sum of HIF-1α and VEGF expression when the cells were cultured with cobalt ions and with dissolution products from the Co-free glass, and was proven to not be due to a rise in pH. The ability of the glass fibres to activate the HIF-1 pathway and promote VEGF expression shows the potential for their use in chronic wound dressings.

## Introduction

Chronic wounds are described as those that do not heal in a timely manner, or that reoccur frequently (Singh et al., 2004; Fonder et al., 2008). When a patient suffers from an underlying pathological condition which impairs healing, such as diabetes, the normal wound healing cascade of hemostasis, inflammation, proliferation, and remodeling can be disrupted. Stalling of healing often occurs in the inflammation phase, resulting in delay of the proliferation phase and wound repair (Boateng et al., 2008; Broderick, 2009). Chronic wounds do not heal through conventional treatment and are often painful, exudating and odorous, which causes a significant reduction in a patient’s quality of life (Franks and Morgan, 2003; Green et al., 2013), and also leads to a huge economic burden to the healthcare system (Sen et al., 2009; Olsson et al., 2019). The number of patients suffering from chronic wounds is expected to grow with an increasingly ageing population and an increased incidence of diabetes (Posnett and Franks, 2008; Frykberg and Banks, 2015).

Healing can be promoted by influencing the hypoxia inducible factor (HIF) pathway, which plays a key role in regulating wound healing and is usually activated under hypoxic conditions (Botusan et al., 2008; Duscher et al., 2015). The HIF-1 pathway is activated through the stabilisation of HIF-1α, which under normal oxygen pressure is constitutively expressed and degraded (Semenza, 2004; Dery et al., 2005). Hypoxia, or hypoxia mimicking agents, stabilise HIF-1α leading to the formation of the HIF-1 complex, which promotes the expression of genes involved in adapting to hypoxia (Gleadle et al., 1995; Semenza, 2004; Esakkimuthukumar et al., 2022). This includes genes that play a role in glucose metabolism and angiogenesis, such as Vascular Endothelial Growth Factor (VEGF). High glucose levels can destabilise HIF-1α in hypoxic conditions, but local stabilisation of HIF-1α has improved wound healing in diabetic mice (Botusan et al., 2008; Zhang et al., 2022a; Zhang et al., 2022b). Wound healing was also impaired in mice with fibroblasts that do not express HIF-1α, which was thought to be due to the decreased expression of VEGF (Duscher et al., 2015). This indicates the HIF pathway could be an important target in developing biomaterials to improve the healing of chronic wounds, particularly in diabetic patients (Zhang et al., 2022a).

Bioactive glasses have recently found clinical application in healing chronic wounds. They were initially investigated for bone repair (Hench et al., 1971; Greenspan, 2019) because they bond to bone *via* formation of a hydroxycarbonate apatite (HCA) layer and they can stimulate bone growth through the release of calcium ions and silica species (Xynos et al., 2001). Due to their amorphous structure, glasses of low silicate content (low network connectivity) can release other ions that can give therapeutic properties, so their use in soft tissue applications is growing (Miguez-Pacheco et al., 2015; Baino et al., 2016; Kargozar et al., 2019), including wound healing (Naseri et al., 2017).

A material for wound healing must be able to be shaped to fit a wound and fibrous scaffolds have been developed for wound healing applications (Han and Ceilley, 2017). Borate-based glass in the form of cotton-like fibres, named MIRRAGEN (ETS Woundcare, Rolla, MO), recently attained FDA approval for chronic wound treatment. The borate glass fibres improved the healing of full thickness wounds in healthy rats (Zhao et al., 2015) and in human clinical trials (Jung et al., 2012). *In vivo*, borate glasses have led to an increase in blood vessels in comparison to silicate fibres when applied to a wound (Zhou et al., 2016) or implanted subcutaneously (Lin et al., 2014), but they also dissolve rapidly, forming HCA. Incorporating copper, which is able to promote angiogenesis, into borate glass fibres further increased VEGF expression and cell migration *in vitro*, and improved wound healing *in vivo* (Zhao et al., 2015). However, the *in vitro* viability and migration of cells exposed to glass fibres is highly dependent on the dose of fibres and the culture conditions (Yang et al., 2015). Recently, sol-gel borate fibres were produced and were found to accelerate the migration of keratinocytes *in vitro* (Naseri et al., 2022).

Silicate glasses may give greater control of dissolution rate and have been investigated for their potential in wound healing (Lin et al., 2012; Yu et al., 2016), but few studies have investigated the effects of silicate glass fibres on wound cells. Here, the aim is to develop fibrous scaffolds of homogeneous thickness that can deliver ions that can stabilise HIF-1α, without promoting mineralisation.

Cobalt is known to stabilise HIF-1α (Yuan et al., 2003) and has been recently incorporated into wound dressings (Shi et al., 2019). Silicate based bioactive glasses containing cobalt (not in the form of fibres) have been produced (Vyas et al., 2015; Kargozar et al., 2018) and been shown to promote VEGF expression by endothelial cells (Quinlan et al., 2015), fibroblasts (Solanki et al., 2021), mesenchymal stem cells (Azevedo et al., 2015), bone marrow stromal cells (Wu et al., 2012; Littmann et al., 2018; Chen et al., 2020), and human umbilical vein endothelial cells (HUVECs) (Kargozar et al., 2017). Cobalt-containing borate glasses also increased HIF-1α, VEGF protein secretion, ALP activity in hBMSC culture (Deng et al., 2019). Cobalt was also incorporated into silicate based sol-gel glass (Barrioni et al., 2018a; Barrioni et al., 2018b; Kermani et al., 2020), provoking VEGF expression from fibroblasts (Dziadek et al., 2018) and HUVECs (de Laia et al., 2021), and into inorganic/organic hybrid scaffolds (de Laia et al., 2020). Interestingly, the original 45S5 Bioglass composition has also been shown increase the expression of VEGF in fibroblasts (Day et al., 2004; Day, 2005; Gorustovich et al., 2010; Gerhardt et al., 2011) and endothelial cells (Leu and Leach, 2008) at specific concentrations. However, the mechanism by which this occurs is not clear. There have been no studies conducted with keratinocytes, which play a crucial role in wound healing.

Fibrous bioactive glass scaffolds can be produced by melt-spinning or electrospinning of the sol-gel process. Electrospun sol-gel glass fibres that only contained silica and calcium oxide in their composition (SiO_2_-CaO) increased VEGF expression from human dermal fibroblast cell line (CD-18CO) (Norris et al., 2020). Melt-derived bioactive glasses of low silicate content often crystallise during conventional fibre drawing or melt-spinning. However, laser spinning has been shown to be a suitable technique to produce fibres of 45S5 Bioglass with mean fibre diameters of around 200–300 nm (Quintero et al., 2009). Here, the aim was to produce laser spun Co-containing bioactive glasses.

Our use of the term “bioactive glass” does not refer to HCA layer formation but rather to a glass that will stimulate a beneficial biological response through its dissolution ions (Rahaman et al., 2011; Jones, 2013). As HCA layer formation is not required for wound healing applications, and apatite deposits have been shown to inhibit the healing of leg ulcers (Tokoro et al., 2009). We previously developed cobalt doped glass compositions designed not form an HCA layer, even in simulated body fluid (SBF) (Solanki et al., 2021). One of these glass compositions (55 mol% SiO_2_, 20 mol% Na_2_O, 10 mol% K_2_O, 10 mol% MgO, 5 mol% CoO) released cobalt into SBF without forming an HCA layer over 21 days. Glasses containing cobalt did stabilise HIF-1α and provoked a significantly higher expression of VEGF in primary human fibroblasts, which was not seen in Co-free controls. This cobalt containing glass composition was chosen for the fibres produced in this study and is referred to as 5Co, and 0Co is the equivalent composition without cobalt (55 mol% SiO_2_, 20 mol% Na_2_O, 10 mol% K_2_O, 15 mol% MgO).

Herein, the hypothesis was that bioactive glass fibres that can deliver cobalt ions, without forming an HCA layer, activate the HIF-1 pathway and therefore have potential for use in a dressing for chronic wounds. The materials were tested using keratinocytes because epidermal VEGF production is required for permeability barrier homeostasis and is thought to stimulate dermal angiogenesis (Elias et al., 2008; Detmar, 2000). In normal human skin, VEGF is expressed and secreted not only by platelets and fibroblasts, but also by epidermal keratinocytes. HIF plays an important role in cytokine production by keratinocytes and in neutrophil recruitment to the skin (Leire et al., 2013). Additionally, HIF-1α is known to stimulate a broad range of effects, and its high level of expression in the basal layer of keratinocytes in the epidermis likely reflects an important role in local and systemic adaptation and sensing to environmental stresses during healing e.g., epidermal oxygenation (Rezvani et al., 2011).

Glass fibres were compared to glass particles to investigate any changes to the glass after processing into fibres. Glass compositions both with and without cobalt were compared to understand if activation of the HIF pathway was solely due to the cobalt released from the glass, or if the other ions released also played a role.

## Materials and methods

### Preparation of bioactive glasses

SiO_2_ (Prince Minerals), CoCO_3_, Na_2_CO_3_, MgO, and K_2_CO_3_ (Sigma Aldrich) were mixed and melted in a platinum-gold crucible for 1.5 h at 1,400°C. Nominal glass compositions both with (5Co) and without cobalt (0Co) are reported in Table 1. The molten glass was quenched into deionised water and the frit was dried overnight at 120°C. Glass plates were prepared by remelting the frit for 30–45 min at 1,400°C and pouring into graphite moulds preheated to 500°C. The plates were left to anneal for 15 min at 500°C before allowing to cool to room temperature. Glass particles were prepared by grinding the frit in a planetary ball mill (Fritsch Pulversitte 7) for 6 min at 500 rpm (no annealing step) to produce glass particles with a D_(0.9)_ of between 60 and 80 μm.

**TABLE 1:** Nominal glass compositions (5Co-Nom and 0Co-Nom) used in this study, and the measured compositions of the glass fibres (5Co-F and 0Co-F) and particles (5Co-P and 0Co-P) in mol% (ICP), showing similar values for the measured and theoretical values. Data are shown as the composition ± maximum error in the ICP-OES measurement.

	*5Co-Nom*	*5Co-P*	*5Co-F*	*0Co-Nom*	*0Co-P*	*OCo-F*
***SiO***_***2***_	55.0	51.4 ± 0.2	56.4 ± 0.3	55.0	47.0 ± 0.2	51.3 ± 0.2
**Na**_**2**_ **O**	20.0	21.6 ± 0.1	19.1 ± 0.2	20.0	23.7 ± 0.1	21.5 ± 0.1
**K**_**2**_ **O**	10.0	11.4 ± 0.0	10.1 ± 0.0	10.0	12.6 ± 0.1	11.3 ± 0.0
**MgO**	10.0	10.6 ± 0.3	9.8 ± 0.2	15.0	16.7 ± 0.2	15.9 ± 0.2
**CoO**	5.0	5.0 ± 0.1	4.6 ± 0.1	0.0	0.0 ± 0.2	0.0 ± 0.0

### Making glass fibres

**FIGURE 1:**
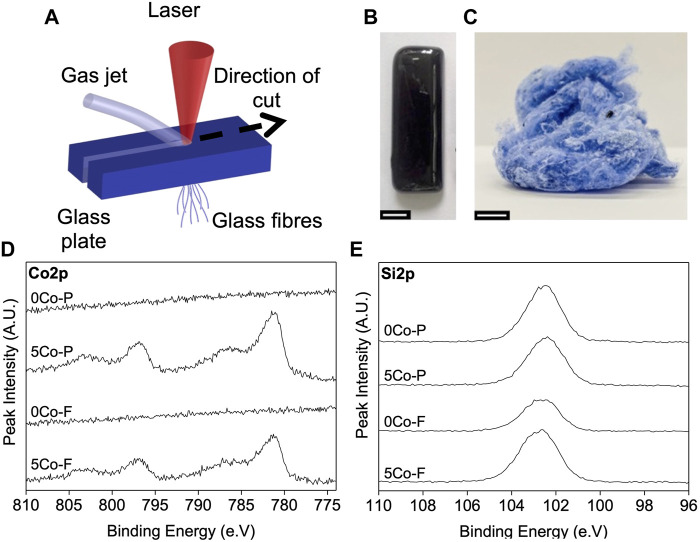
**(A)** Schematic of the laser spinning process using a glass precursor plate **(B)** photograph of an example of a bioactive glass precursor plate (scale bar is 1 cm) **(C)** mesh of fibres produced **(D)** XPS core line spectra of cobalt (Co2p) showing no difference in oxidation state of Co between the cobalt glass fibres and particles, and the core line spectra of silica (Si2p) **(E)** showing no difference in oxidation state of Si between the 5Co or 0Co glass fibres (5Co-F and 0Co-F) or particles (5Co-P and 0Co-P).

Figure 1A shows a schematic of glass fibre (Figure 1C) production from the glass plates (Figure 1B) by laser spinning, as described previously (Quintero et al., 2009; Quintero et al., 2014). A high-power laser beam is used to melt a small volume of a precursor glass monolith. At the same time, a supersonic gas jet is applied to elongate the molten material by the action of the viscous friction at the gas/melt interface. This jet also produces the fast cooling and solidification of the fibres, hence producing amorphous nanofibers during the process. A CO_2_ laser (Rofin Sinar DC035) was used, emitting with wavelength of 10.6 μm and radiant power of 2.5 kW. The laser beam was focused 13 mm above the surface of the glass plate using a lens with focal length of 190.5 mm. Compressed air was supplied to the co-axial nozzle and the off-axis supersonic nozzle at a pressure of approximately 5 and 10 bar, respectively. The plate was moved with velocity relative to the laser beam of 300 mm min^−1^ to produce the glass fibres.

### Measuring glass compositions

The composition of the glass fibres and particles was measured by Inductively Coupled Plasma Optical Emission Spectroscopy (ICP-OES) (Thermo Scientific, iCAP6000 Series ICP). 100 mg of glass fibres or particles were mixed with 400 mg lithium metaborate (Alfa Aesar) and heated to 950°C for 30 min in a platinum crucible to form a fused pellet. Once cooled, the pellet was dissolved in 50 ml of 2 M HNO_3_. This was diluted 1:10 and ICP-OES used to measure the elemental concentration of the solution, and the composition calculated.

### Ion release into DMEM

Glass fibres and particles of both compositions were incubated in Dulbecco’s Modified Eagle Medium (DMEM, LifeTechnologies) at a ratio of 1.5 mg ml^−1^ to evaluate the ion release (Macon et al., 2015). 10 ml DMEM was added to 15 mg of fibres or particles and incubated at 37°C in an orbital shaker under agitation at 120 rpm. At each time point (30 min, 1, 2, 4, 8, 24 h, 3, and 7 days) 0.5 ml of DMEM was collected and diluted 1:20 with deionised water to quantify the ionic concentration by ICP-OES, and replaced with 0.5 ml of fresh DMEM. At 7 days, samples were collected using filter paper, rinsed with deionised water and acetone, and dried overnight.

Fourier Transform Infrared Spectroscopy (FTIR), X-ray diffraction (XRD) and Scanning Electron Microscopy (SEM) were conducted on fibres and particles, before and after incubation in DMEM. FTIR spectra were collected with a Nicolet iS10 FTIR in Attenuated Total Reflectance mode from 400–2000 cm^−1^ with a resolution of 0.4 cm^−1^. XRD was carried out on a PANalytical XRD measuring between 5 and 80° 2θ using a step size of 0.0334225. SEM images were taken after coating samples in chromium, using a LEO Gemini 1525 FEGSEM and an accelerating voltage of 5 kV.

### XPS

Surface compositions and chemical states were obtained after drying the samples at 120°C for 1 h and conducting X-ray photoelectron spectroscopy (XPS) using a Thermo K Alpha + spectrometer (Thermo Scientific) operating at around 10^−8^ to 10^−9^ Torr with a Al Kα radiation source (1,486.7 eV). High resolution spectra were collected for cobalt (Co 2p) and silica (Si 2p) to assess the oxidation states with the peaks normalised to the carbon peak (285.0 eV).

### FIB/TEM

Samples were prepared using a focussed ion beam (FIB) operated at 30 kV (FIB, FEI Helios NanoLab 600) for analysis by Transmission Electron Microscopy (TEM) and Energy Dispersive Spectroscopy (EDS). Briefly, a 15 μm × 2 μm site was coated with 1.5 μm of platinum. Two trenches were made on either side of the platinum layer and the base of the region was cut, using currents of 2.8 and 6.4 nA respectively. The sample was attached to the omniprobe manipulator, lifted out, and attached to a TEM lift-out three post copper grid (Agar Scientific). Samples were thinned to a width of around 100 nm using currents between 0.46 and 2.8 nA. Finally, the sample was polished with a gallium ion beam operated at 2 kV to remove possible artefacts introduced by milling. The FIB samples were imaged in the TEM (JEOL JEM 2100F) operating at 200 kV using dark field Scanning Transmission Electron Microscopy (STEM) mode. Elemental maps were obtained by EDS (Oxford Instruments INCA EDS 80 mm X-Max detector system with light-element (Z > 5) analysis and STEM capability).

### Cell culture

Immortalised human epidermal keratinocyte (HaCaT; RRID:CVCL_0038) cells were cultured in DMEM, supplemented with 10% Fetal Bovine Serum (FBS), and maintained in an incubator kept at 37°C and 5% CO_2_, which was also used as control medium. The cell line present in this study were obtained from Dr Vania Braga, Imperial College London, who has curated the cells since obtaining the cell line from the group of NE Fusenig, German Cancer Research Center, Heidelberg, Germany, who first isolated and characterised the cells, demonstrating their full epidermal differentiation capacity (Boukamp et al., 1988).

Bioactive glass conditioned medium was prepared immediately before use. Glass fibres were sterilised by dry heat at 120°C for 2–3 h and incubated with DMEM (without serum) at a ratio of 6.67 mg ml^−1^. The medium was conditioned with glass fibres for 24 h, after which it was removed, sterile filtered through a 0.2 µm syringe filter and transferred to T25 flasks with vented caps. The conditioned medium was kept an incubator at 37°C with 5% CO_2_ overnight to allow equilibration of the pH prior to use. Conditioned medium from the glass particles was made in the same way, but the particles were not sterilised at 120°C. For CoCl_2_ controls, a 10 mM stock solution of CoCl_2_ was made up in distilled water and added to DMEM to give a final concentration of 300 µM before equilibrating the pH. When producing conditioned medium from either the glass fibres or glass particles, five groups were investigated: 1—DMEM control; 2—DMEM +300 μM CoCl_2_; 3—5Co conditioned DMEM; 4—0Co conditioned DMEM; and 5—0Co conditioned media +300 µM CoCl_2_. When investigating the high pH media, NaOH was added dropwise to DMEM to give a pH of 8.5 and 9.5, after which the media was sterile filtered and the pH allowed to equilibrate overnight before use in T25 flasks with vented caps.

### In-cell western for HIF-1α

To determine the amount of HIF-1α after exposure to conditioned media, 4 × 10^4^ cells were seeded per well of a 48 well plate and left for 2–3 days to reach confluence. The medium was removed, and wells washed once with PBS before adding 400 µL conditioned medium to each well. After 4 h, the conditioned medium was removed, wells washed with PBS and cells fixed in a 3.7% formaldehyde solution. An In-Cell Western blotting (ICW) was conducted using a protocol modified from Cell Signalling Technology. Briefly, wells were permeabilised with 0.25% Triton 100X for 5 min, washed, and blocked with blocking buffer (3% w/v Bovine Serum Albumin (BSA) in PBS/0.1% v/v Tween 20) for 1 h. The mouse monoclonal primary antibody for HIF-1α (Abcam, ab16066) was diluted 1:400 in blocking buffer and incubated for 1.5 h at room temperature. A donkey anti-mouse IR secondary antibody (Licor, 925–32212) and DRAQ5 (New England Biolabs, 4084S) were diluted 1:2000 in blocking buffer and incubated for 1 h, before the plate was read using the Licor Odyssey.

### ELISA for VEGF

To measure the amount of VEGF secreted by cells exposed to conditioned media, 1 × 10^4^ cells were seeded per well of a 96 well plate and left for 1 day to attach. Conditioned media was supplemented with 10% FBS prior to equilibrating the pH, and 100 µL was added to each well. This was changed for serum free conditioned media 24 h before collecting the supernatant and cell lysate at 1, 3 and 7 days. Supernatants were kept at −80°C before an ELISA was carried out using a human VEGF antibody pair kit (LifeTechnologies, CHG0113) according to the recommended protocol, and a plate reader (Perkin Elmer Enspire) used to measure the absorbance at a wavelength of 450 nm with a reference wavelength of 650 nm. Cell lysates were prepared by collecting the cell monolayer in 200 µL 1 v/v% triton 100X in PBS, and freeze thawing 3 times. The DNA was quantified from the cell lysates using a florescent dye which binds DNA (bisbenzimidazole, Hoechst 33258), with a standard curve prepared using a DNA standard (LifeTechnologies). The fluorescence was measured at 360 nm excitation and 460 nm emission using a plate reader (Perkin Elmer Enspire).

### Nanoparticle measurement in the conditioned media

While the glass particle and glass fibre conditioned medium was expected to contain ionic dissolution products of the glasses, nanoparticles may also have been lost from the glasses and therefore given to the cells. The concentration and size distribution of any nanoparticles in the conditioned media was therefore measured using a Nanosight NS300 (Malvern). Conditioned media was diluted in fresh, confirmed particle-free 18.2 MΩ water until the concentration of particles was within the linear range of the instrument (1–10 × 10^8^ particles ml^−1^). Five 60-s measurements were taken per sample at Camera Level 15 and Detection Threshold 5. The FTLA (Finite Track Length Adjusted) size distribution smoothing algorithm was disabled to avoid generation of artefact peaks.

### Statistics

All results are expressed at the mean ± standard deviation. Statistical significance between the different groups was determined using a one or two way ANOVA and Tukey’s test.

## Results

### Producing glass fibres

Glass fibres were successfully formed by laser spinning, which produced an intertwined mesh of fibres (Figure 1C), with diameters ranging from ∼0.5 μm—10 μm. As shown in Table 1, the compositions of the fibres (5Co-F and 0Co-F) as measured by ICP-OES were close to the measured compositions of the glass particles (5Co-P and 0Co-P) and to the nominal compositions. This is in agreement with previous work which showed that the laser spinning process does not significantly change the composition of bioactive glasses (Quintero et al., 2009).

### XPS analysis of fibres

XPS was conducted to determine if the oxidation states of cobalt or silica varied between the glass fibres and glass particles. The core line spectra of cobalt (Co2p) is shown in Figure 1D, and is comprised of a spin split orbital associated with a shake-up satellite. The spin split orbital is between 781.13 and 781.28 eV for Co2p 3/2, and 796.83–796.98 eV for Co2p 1/2 with splitting energy at 15.7 eV. The shake up satellites are between 786.33—786.78 eV and 802.73—802.98 eV (Ho et al., 1990; Tan et al., 1991; Khodakov et al., 2002; Zhang et al., 2009). The core line spectra of Si2p are shown in Figure 1E and the peak at 102.6 ± 0.3 eV is attributed to Si^4+^ (Barr, 1983). There was little difference between the fibres and the particles, suggesting that the oxidation state of both cobalt and silica are unchanged by the laser spinning process.

### Ion release into DMEM medium

**FIGURE 2:**
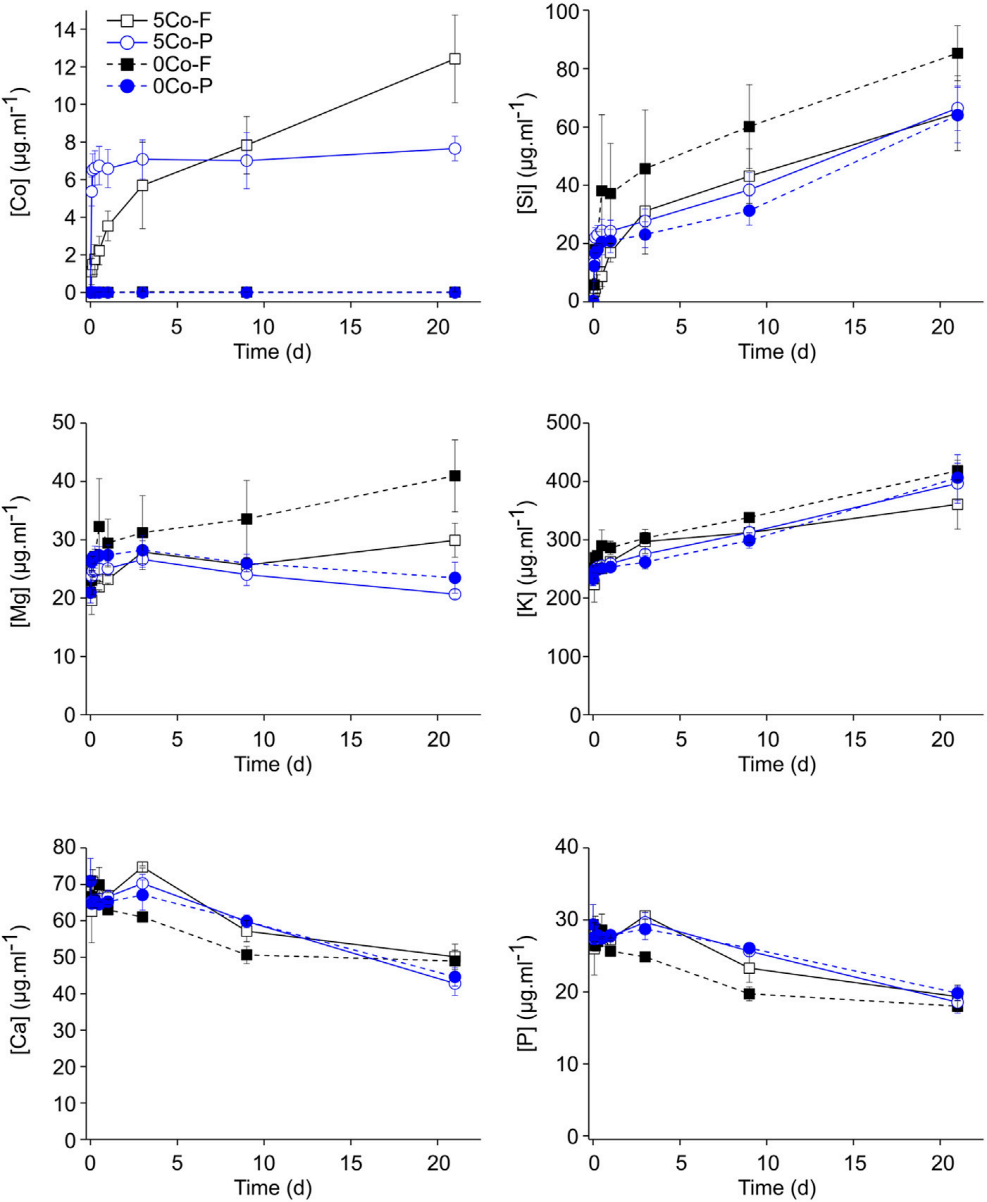
Elemental concentration in DMEM after incubation with 5Co and 0Co glass fibres (5Co-F and 0Co-F) and particles (5Co-P and 0Co-P) at a concentration of 1.5 mg ml^−1^ as a function of time of immersion. Data is represented as the mean ± standard deviation with N = 3.

The ion release from the glass fibres and particles of 5Co and 0Co into DMEM over 7 days is shown in Figure 2. The 5Co-P gave a burst release of cobalt in the first 30 min, and by 1 h the cobalt concentration reached a plateau between 5.7 and 8.5 μg ml^−1^. A burst release of cobalt was not seen from 5Co-F, instead there was a continuous increase for up to 7 days without a plateau being reached. At 24 h, the Co release from the fibres and particles was similar, but by 7 days the Co concentration reached approximately 12 µg ml^−1^ for 5Co-F, compared to around 7.5 µg ml^−1^ for 5Co-P. A burst release of Co was observed in the particles because fluid was accessible all around the particles, allowing rapid ion exchange (Co ions for H^+^ in the DMEM) and formation of the silica rich layer. In the fibres, ion exchange was initially slower than the particles, but release was more sustained as the distance for Co ion diffusion was less than for the particles, due to the fibre thickness being at least an order of magnitude smaller than the particle diameters.

At 30 min, the concentration of the silica released from the fibres was lower than that of the glass particles, with around 5 μg ml^−1^ measured for the fibres, and around 13 μg ml^−1^ for the particles, but by 8 h the silica release from the fibres and particles was similar. For up to 7 days, there was a steady increase in the amount of silica released without a plateau being reached, and the final concentration was between 52 and 80 μg ml^−1^.

For both the particles and fibres in both compositions, the concentration of calcium and phosphate decreased in a similar manner. At 24 h, the concentration remained steady at around 65–70 μg ml^−1^ for calcium and approximately 27 μg ml^−1^ for phosphorous. After this time, a decrease in both calcium and phosphorus was measured for all samples, suggesting that there was some precipitation of calcium phosphate on the glass surface.

### Characterisation of the glasses after incubation in DMEM media

**FIGURE 3:**
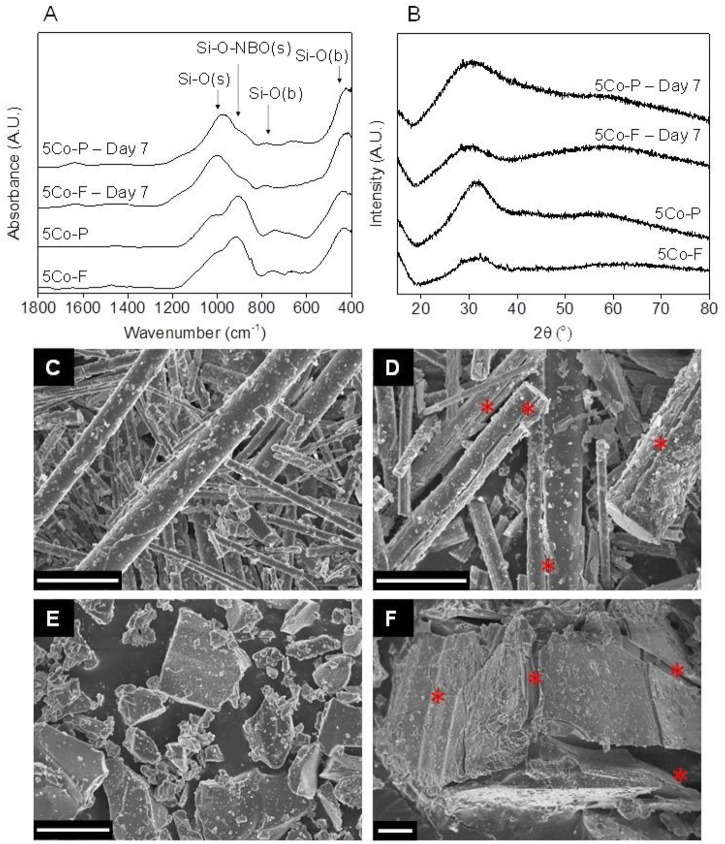
FTIR **(A)** and XRD **(B)** of the glasses containing cobalt before and after incubation in DMEM for 7 days: 5Co fibres (5Co-F) and particles (5Co-P); SEM images of 5Co-F before **(C)** and after **(D)** incubation in DMEM for 7 days, and 5Co-P before **(E)** and after **(F)** incubation in DMEM at a concentration of 1.5 mg ml^−1^. Scale bar is 10 μm, and * indicates cracks forming in the fibres and particles after incubation in DMEM.

XRD and FTIR of the glass fibres and particles (Figure 3; Supplementary Figure S1) showed that glass with (Figure 3) and without cobalt (Supplementary Figure S1) behaved in a similar manner. Prior to incubation in media, FTIR spectra obtained from the particles and fibres looked similar with bands at approximately 1,000, 920 and 450 cm^−1^ corresponding to the Si-O(s), the Si-O(s) associated with a modifying cation, and the Si-O(b) respectively (Sanders et al., 1974; Serra et al., 2003; Liu et al., 2013).

After incubation in medium, no differences were seen between the glass fibres and particles. The FTIR spectra showed a decrease in the Si-O(s) associated with a modifying cation, as the modifiers left the glass and accumulated in the medium (Filgueiras et al., 1993), as measured by ICP-OES (Figure 2). The band corresponding to the Si-O(s) at around 1,000 cm^−1^ became more prominent, suggesting the formation of a silica rich layer (Serra et al., 2003). All samples were amorphous both before and after incubation in DMEM for 7 days, but in the cobalt containing samples the XRD patterns showed a second amorphous halo at higher values of °2θ. There was no evidence of HCA formation.

SEM images in Figure 3D and SI Supplementary Figure S1D show how the glass fibres and particles degraded. Prior to incubation (Figure 3C and SI Supplementary Figure S1C), the fibres of both 0Co and 5Co looked similar in morphology. The fibres had a wide range of diameters, and some particulates were seen adhering to the glass fibres which were due to glass dust forming during the laser spinning process. After incubation in DMEM for 7 days, no difference was seen between the 5Co (Figure 3D) and 0Co (Supplementary Figure S1D) compositions. For both 5Co-F and 0Co-F, a thin layer of precipitate was present and many of the fibres had cracked, as indicated in Figure 3D. Similar cracks have been reported to be due to the formation of a silica rich layer (Dietrich et al., 2009). The precipitated layer was seen to have peeled away from the fibre, revealing an underlying smoother surface, and often showed that the fibre was cracked in multiple places through the cross section. A precipitated layer was also seen on the glass particles after incubation in media. Most of the particles had cracked, and although the precipitated layer was observed to come away from the particles, this occurred less frequently compared to the fibres.

### Elemental analysis of glasses by TEM

**FIGURE 4:**
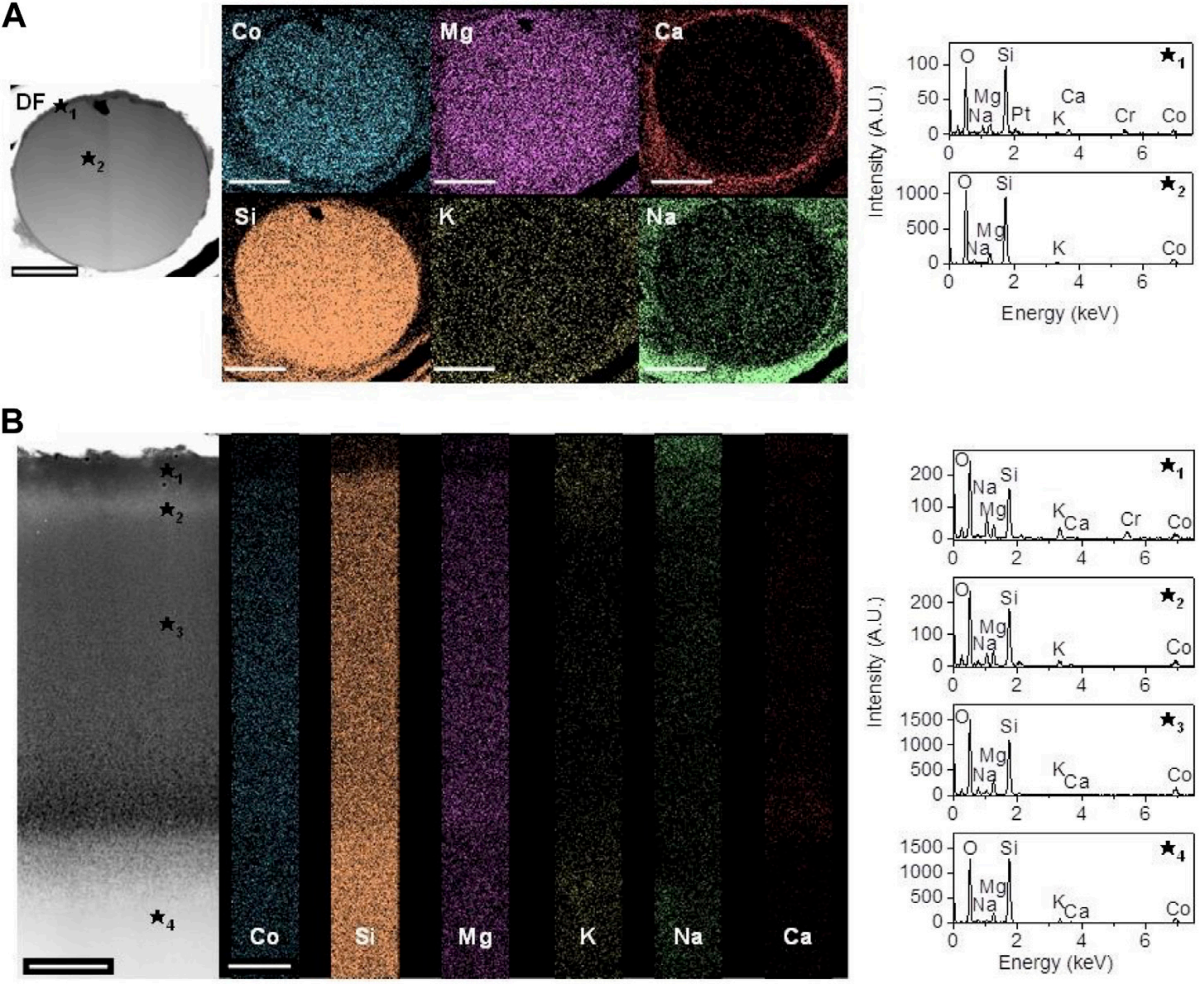
Darkfield TEM images and EDX spectra of a 5Co fibre cross section **(A)** and 5Co particle **(B)** showing several reaction layers with differing elemental concentrations after incubation in DMEM for 7 days. Scale bar for **(A)** is 1 µm for **(B)** is 250 nm.

TEM-EDS is an important tool for understanding nanoscale changes to glass surface during degradation. SEM images showed little differences between 5Co or 0Co glasses and ion release was similar, except for the release of cobalt. Therefore, the 5Co composition was chosen to assess the changes to the glass fibres and particles at the nanoscale using TEM-EDS (Figure 4). Prior to incubation in DMEM, the fibres and the particles did not show any reaction layers and were free of precipitate (Supplementary Figure S2). After 7 days in DMEM (Figure 4A), a layer of precipitate was seen around the fibres, as seen under the SEM, and it bridged a crack in the glass fibre, suggesting that, for some fibres at least, the precipitated layer formed before the fibre cracks. EDS of the precipitated layer showed that it contained calcium, phosphate, magnesium, cobalt and sodium (higher resolution image shown in Supplementary Figure S3). Elemental mapping of the cross-section of the fibres showed homogeneous distribution of Si, Co and Mg throughout, with no Ca, and reduced concentration of Na and K towards the glass surface, due to dissolution (ion exchange).

Figure 4B shows several reaction layers close to the top surface of a glass particle, after incubation in DMEM for 7 days, with the top layer containing similar elements as the precipitated layer on the fibres. Subsequent layers had a similar composition to each other and to the centre of the glass fibres. Although the relative composition of the glass particle was consistent throughout the depth, except for the top layer, there was a clear difference in the contrast of the particle between layers ★_3_ and ★_4_, with layer ★_3_ appearing to be much darker, which is indicative of a lower density. This observation was not made for the glass fibres. K and Na were detected on the surface of both the glass fibres and glass particles, indicating that there may also be small amounts of residual salts from the DMEM media, such as NaCl and KCl present on the glass surface.

### HIF-1α stabilisation and VEGF expression of fibres

**FIGURE 5:**
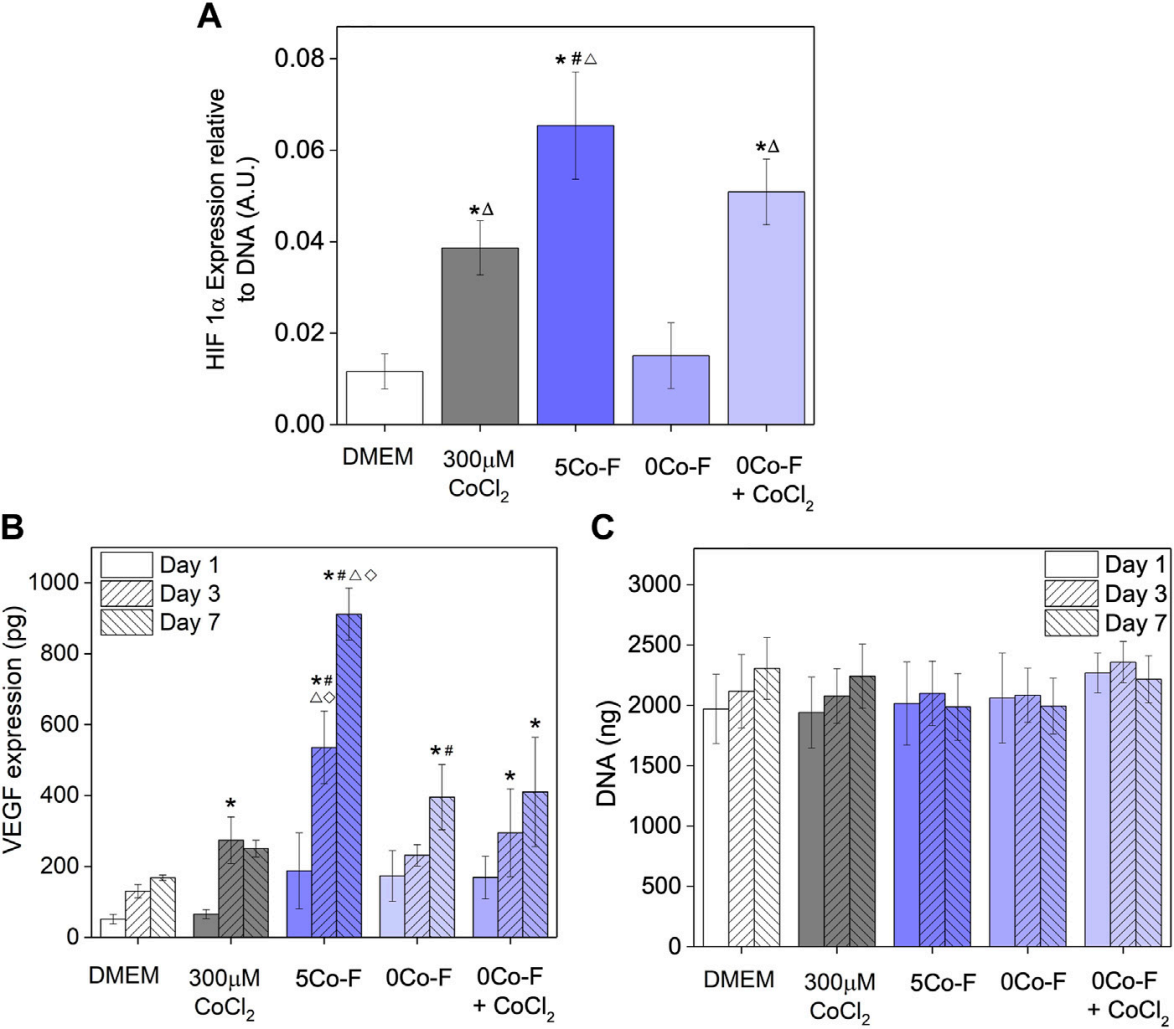
Results of *in vitro* studies of keratinocytes exposed to conditioned media from the glass fibres: Increase in HIF-1α measured after 4 h **(A)**; VEGF expression **(B)**; and total DNA **(C)**. Data is represented as mean ± standard deviation with N = 4. For Fig B and C the 0Co-F + CoCl_2_ condition is represented as N = 3 for Day 1 and 3, and n = 2 for Day 7. Statistical significance was determined with a **(A)** one way ANOVA or **(B)** and **(C)** two way ANOVA, with a Tukey means comparison test with *p < 0.05 compared to DMEM, #*p* < 0.05 compared to 300 μM CoCl_2_, Δ *p* < 0.05 compared to 0Co-F and ⋄*p* < 0.05 compared to 0Co-F + CoCl_2_.

To determine whether the glass conditioned media could activate the HIF pathway in cells that are known to play a key role in wound healing, keratinocytes were used to measure the stabilisation of HIF-1α and expression of VEGF protein (Figure 5). Media contained either cobalt from cobalt chloride or as dissolution products of the glasses. Significantly higher HIF-1α was measured after keratinocytes were exposed to cobalt containing media when compared to the DMEM only control, regardless of whether the cobalt was added as CoCl_2_ or released from 5Co-F. Surprisingly, the amount of HIF-1α measured after exposure to 5Co-F conditioned medium was significantly higher than that produced by cells exposed to media with 300 μM CoCl_2_, even though the amount of cobalt present in the media was similar (17.0 ± 0.6 μg ml^−1^ for 300 µM CoCl_2_ and 14.7 ± 1.2 μg ml^−1^ for 5Co-F). To determine whether the cobalt ions released from the glass fibres had the same effect as adding CoCl_2_ to the dissolution products of a cobalt free glass, the effect of 0Co-F conditioned media supplemented with 300 µM CoCl_2_ was also investigated. Importantly, HIF-1α measured in cells incubated with 0Co-F + CoCl_2_ did not match that of cells incubated with 5Co-F, even though their Co concentration in solution (measured by ICP) was similar.

The expression of VEGF showed a similar trend to the HIF-1α measurements. For all conditions, an increase in VEGF expression was seen between Day 1 and 7. The VEGF expression was significantly higher for CoCl_2_ and all glass conditioned media compared to the DMEM control. Incubating cells in 5Co-F conditioned medium led to a significantly higher VEGF release compared to 300 μM CoCl_2_; and incubating cells in 0Co-F + CoCl_2_ did not replicate the effect of 5Co-F: VEGF protein expression in the 0Co-F + CoCl_2_ group was found to be significantly lower than that of 5Co-F treatment but significantly higher than the 300 µM CoCl_2_ condition. The only difference in ion concentration between the two groups was elevation Mg and Si concentration in 0Co-F + CoCl_2_ compared to 5Co-F conditioned media (Figure 2). The DNA measured for all conditions was similar, with no significant differences observed with time or between conditions, indicating that the differences in protein expression were not due to changes in cell number.

### Investigating the effect of conditioned media from 5Co-F

**FIGURE 6:**
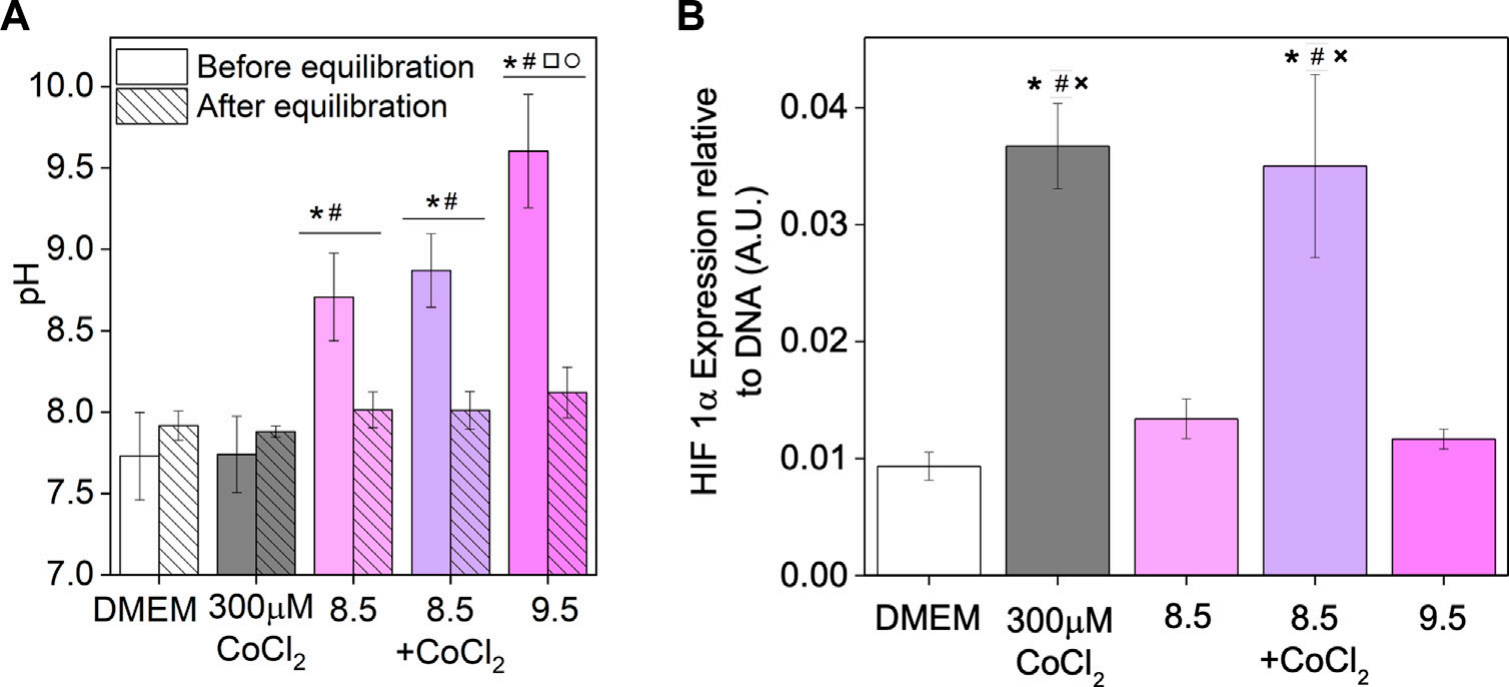
Effect of pH on HIF-1α expression: pH of DMEM media before and after equilibration overnight in a 5% CO_2_ incubator **(A)** and HIF-1α measured when exposing keratinocytes to media of elevated pH of 8.5 (with and without cobalt chloride) and 9.5 after equilibration overnight **(B)**. Data is represented as mean ± standard deviation with N = 3. Statistical significance was determined with a **(A)** two way ANOVA or **(B)** one way ANOVA, with a Tukey means comparison test with *p < 0.05 compared to DMEM, #*p* < 0.05 compared to 300µM CoCl_2_, □ *p* < 0.05 compared to 8.5, ○ *p* < 0.05 compared to 8.5 + CoCl_2_ and ✕*p* < 0.05 compared to 9.5.

Investigating the HIF-1α stabilisation and VEGF expression showed that conditioned medium from 5Co-F produced an effect that could not be replicated by an equivalent amount of CoCl_2_ or by supplementing 0Co-F conditioned medium with CoCl_2_. As glass dissolution can cause pH increase, through ion exchange (e.g. Na^+^ of the glass for H^+^ from the medium), a pH change could also have an effect on the *in vitro* cellular response in addition to the ionic concentration of conditioned medium. The pH of DMEM increased after incubation with bioactive glasses (Figure 6) to a maximum value of 9.5 and was allowed to equilibrate overnight, but a slight increase in pH was still seen in the bioactive glass conditioned medium. Therefore, the effect of an elevated pH on HIF-1α was investigated. The pH of the medium was increased to pH 8.5 and 9.5, using NaOH, to mimic the burst rise seen when incubating bioactive glasses in cell culture medium, and the effect of an increased pH in combination with CoCl_2_ was also investigated as shown in Supplementary Figure S4. After equilibration of the high pH media in a 5% CO_2_ incubator overnight, the pH decreased to levels similar to the DMEM and CoCl_2_ controls. Although the measured pH was always slightly higher than the controls, the difference was not statistically significant. After allowing the pH to equilibrate overnight, keratinocytes were incubated in the medium and the HIF-1α measured, as shown in Figure 6B. Only the presence of CoCl_2_ led to an increase in HIF-1α and no difference was seen between the addition of CoCl_2_ and addition of CoCl_2_ in combination with pH adjusted to a higher value prior to overnight equilibration.

**FIGURE 7:**
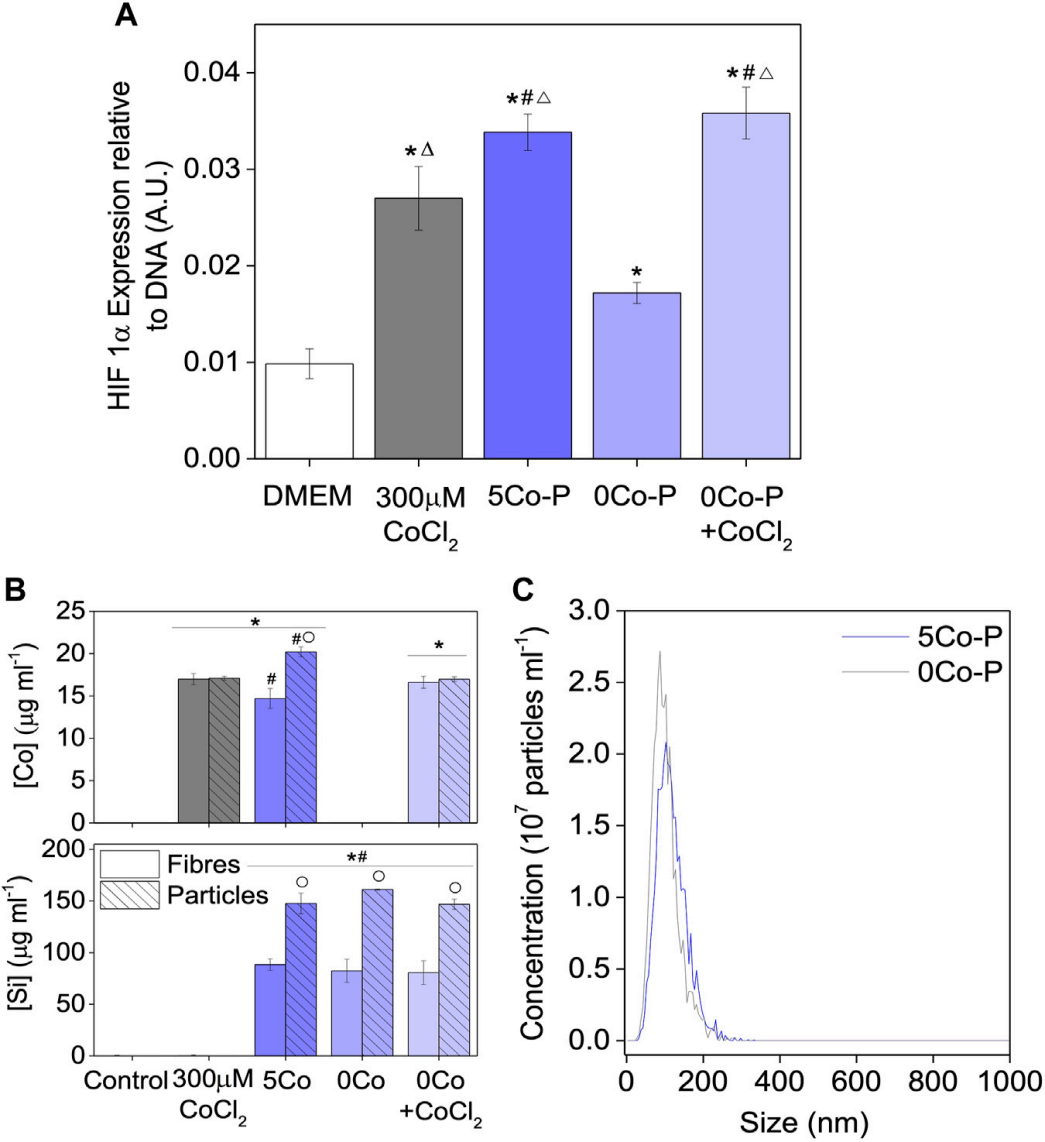
Increase in HIF-1α measured when keratinocytes are exposed to conditioned media from glass powder for 4 h. Data are represented as mean ± standard deviation with N = 3 **(A)**. Co and Si concentrations in DMEM for the glass fibre and glass particle conditioned media prepared at a ratio of 6.67 mg ml^−1^; data are represented as mean ± standard deviation with N = 7 for the Fibre conditions and N = 3 for the Particle conditions **(B)** and size distribution of nanoparticles measured, by Nanoparticle Tracking Analysis, in 5Co and 0Co glass particle conditioned media (5Co-P and 0Co-P) **(C)**. Statistical significance was determined with a one way ANOVA, with a Tukey means comparison test with *p < 0.05 compared to DMEM, #*p* < 0.05 compared to 300 μM CoCl_2_, Δ *p* < 0.05 compared to 0Co-P in **(A)** and *p < 0.05 compared to DMEM in both the fibre and particle experiment, #*p* < 0.05 compared to 300 µM CoCl_2_ controls in both the fibre and particles experiment, ○ *p* < 0.05 compared to the glass particle conditioned media for the same condition in **(B)**.

HIF-1α was also measured in cells incubated with conditioned media from the glass particles (5Co-P and 0Co-P, Figure 7A) to understand whether the effects of 5Co-F were due to the glass composition, or differences between the fibres and particles. As seen with the glass fibre conditioned media, an increased amount of HIF-1α was measured with 300 μM CoCl_2_, 5Co-P, and 0Co-P + CoCl_2_ when compared to the DMEM control, with 5Co-P being significantly higher than 300 µM CoCl_2_. However, unlike with the glass fibre conditioned media, the amount of HIF-1α measured for 0Co-P + CoCl_2_ was the same as 5Co-P. A smaller significant increase in HIF-1α was also measured for the 0Co-P conditioned media compared to DMEM, which was not seen in the conditioned medium prepared with glass fibres.

The elemental concentration of the conditioned media from both the fibres and particles was characterised by ICP-OES and the Si and Co. concentrations are shown in Figure 7B. The 5Co-P medium contained a slightly higher concentration of cobalt (20.2 ± 0.6 μg ml^−1^) compared to in the 5Co-F conditioned medium (14.7 ± 1.2 μg ml^−1^). The glass particle conditioned media also contained higher concentrations of magnesium, and calcium and phosphorous, as seen in Supplementary Figure S4. This suggests there may have been less precipitation of a cobalt and magnesium substituted calcium phosphate layer on the glass particles after 24 h, compared to the glass fibres. The most striking difference observed was the difference in the amount of Si, with a concentration of around 150 μg ml^−1^ Si measured for the glass particle conditioned media compared to approximately 80 μg ml^−1^ in the glass fibre conditioned media. A concentration of 150 μg ml^−1^ silica in solution is above the reported solubility limit in water (116 μg ml^−1^ at 25°C (Gunnarsson and Arnorsson, 2000)). When the solubility limit is exceeded, silica can polymerise and form nanoparticles, although this is highly dependent on the pH, temperature and ionic strength of the aqueous solution (Tobler et al., 2009). The presence of nanoparticles was investigated, using Nanoparticle Tracking Analysis, to try and explain the high concentration of silica measured by ICP-OES. Nanoparticles were detected in the glass particle conditioned media, for both 5Co and 0Co, and the size distribution of these particles is shown in Figure 7C. Nanoparticles in the 5Co-P conditioned media had a modal size of 117 ± 6 nm and the concentration of particles was 8.5 ± 0.9 × 10^9^ ml^−1^, whereas for 0Co-P conditioned media the modal size was 102 ± 4 nm and concentration was 9.2 ± 1.0 × 10^9^ ml^−1^. In contrast, a reliable measurement of nanoparticles could not be obtained from the glass fibre conditioned media, for either 5Co or 0Co, or the DMEM control, as the concentration was below the detection limit of 5 × 10^7^ particles ml^−1^.

## Discussion

The aim of this study was to investigate the use of glass fibres as a device to activate the HIF pathway and therefore promote the healing of chronic wounds. The aim would be to use the fibres to fill a wound, which would then be covered with a conventional dressing. The fibres would be a temporary scaffold for wound regeneration biodegrading and releasing their active ions. The materials properties of both fibres and particles, with and without cobalt, were compared and the *in vitro* response of keratinocytes to conditioned media from the glasses was characterised.

The fibre spinning process retained the amorphous structure of the glasses (Figure 3). The ion release characteristics during incubation in DMEM for the glass fibres and particles, at a concentration of 1.5 mg ml^−1^, either with or without cobalt, were similar (Figure 2), indicating that the laser spinning process did not significantly change the glass. After incubation in DMEM, a calcium phosphate layer containing cobalt and magnesium formed on the surface of the fibres and particles (Figure 4; Supplementary Figure S3) but it was not crystalline (Figure 3). Cobalt has previously been shown to substitute into calcium phosphate on bioactive glass (Hoppe et al., 2014), and both cobalt and magnesium have previously been seen to substitute into the calcium phosphate layer when incubating 5Co particles in SBF for 21 days (Solanki et al., 2021). When extracts were prepared for cell culture, ion release was higher for particles compared to the fibres (Figure 7). The conditioned media were prepared with a higher concentration of glass (6.67 mg ml^−1^) in the media, compared to the degradation study (Figure 2). The increased Si content in the media conditioned with the glass particles (150 mg ml^−1^), compared to that prepared with the fibres (80 mg ml^−1^) and the subsequent discovery of nanoparticles (mean of 98–123 nm) was unexpected and highlights the need for proper analysis of conditioned media before carrying out cell culture. The glass fibre conditioned media did not contain any nanoparticles in a measurable concentration.

The *in vitro* cell response to bioactive glasses that contain Co has previously been characterised in mesenchymal stem cells (Azevedo et al., 2015), bone marrow stromal cells (Wu et al., 2012), osteoblasts, fibroblasts (Solanki et al., 2021) and endothelial cells (Quinlan et al., 2015; Solanki et al., 2021). Cobalt is known to stabilise HIF-1α in environments with normal oxygen levels (Yuan et al., 2003), and cobalt doped bioactive glasses have been shown to cause an increase in proteins such as VEGF (Wu et al., 2012; Azevedo et al., 2015; Quinlan et al., 2015) which can promote angiogenesis. Here, the *in vitro* cell response of keratinocytes to conditioned media from cobalt doped bioactive glasses was investigated for the first time, as these cells are crucial in the wound healing process. In the current study, incubating keratinocytes in 5Co-F conditioned media led to a significantly higher measurement of HIF-1α compared to the equivalent amount of CoCl_2_ (Figure 6). When adding CoCl_2_ to 0Co-F medium, the response did not match that of 5Co-F, suggesting there is a synergistic effect of the glass dissolution products which is more than a simple combination of ions released from the glass and cobalt from CoCl_2_. The trends seen in the measurement of HIF-1α were reflected in the VEGF expression. 5Co-F conditioned media caused a significant increase in VEGF expression when compared all other conditions, and this effect could not be replicated by the addition of CoCl_2_ to 0Co-F conditioned media. Interestingly, 0Co-F caused an increase in VEGF expression, even though cobalt was not present, and an increase in HIF-1α was not measured with this condition. The ability of bioactive glasses such as 45S5, which do not contain cobalt, to cause an increase in VEGF production has previously been reported in fibroblasts (Day et al., 2004; Day, 2005; Gerhardt et al., 2011) and endothelial cells (Leu and Leach, 2008), but the mechanism by which it occurs is not clear (Ranmuthu et al., 2020). The effect is attributed to silicon and calcium ion release, for example conditioned media from bioactive glass nanoparticles of SiO_2_-CaO-P_2_O5 compositions stimulated up-regulated expression of the VEGF, basic fibroblast growth factor, their receptors, and endothelial nitric oxide synthase from HUVECs, resulting in enhanced tube formation *in vitro* (Mao et al., 2015). Shi *et al.* found that bioactive dissolution ions significantly promoted the VEGF production from cardiomyocytes, which up-regulated the HIF-1α pathway, which then mediated the behaviour of endothelial cells (Shi et al., 2021). This previous work therefore supports the evidence of synergy between Si, Ca and Co ions on VEGF production.

For the first time, a significant difference has been shown in the *in vitro* cellular response to conditioned media from a glass releasing pro-angiogenic ions compared to an equivalent amount of the ions in media, or conditioned media from an un-doped glass supplemented with the therapeutic ions. The effect of 5Co-F on HIF-1α and VEGF expression could not be replicated by the equivalent amount of CoCl_2_ in DMEM or by the addition of CoCl_2_ to 0Co-F conditioned media. To gain a further understanding of this effect, the pH was investigated. When bioactive glasses are incubated in aqueous buffer, the pH rises as modifying cations are exchanged with H^+^ from the solution (Hench, 1991). Although the pH of the glass conditioned media was left to equilibrate overnight immediately prior to incubation with the cells, the pH was still slightly more basic for all glass conditioned media compared to the DMEM and CoCl_2_ controls (Supplementary Figure S4). Measurement of HIF-1α after exposing keratinocytes to media adjusted to high pH and left to equilibrate overnight, with or without CoCl_2_, showed no effect of incubating keratinocytes in CoCl_2_ at a slightly basic pH, showing that the higher HIF-1α and VEGF measured with 5Co-F was not due to the combination of cobalt and an increased pH.

Finally, HIF-1α was measured after incubating keratinocytes with conditioned media from the glass particles, to understand if the synergistic effect observed was due to the glass composition or processing into glass fibres. Figure 7A shows a significantly higher amount of HIF-1α was measured when incubating cells 5Co-P conditioned media compared to DMEM with only CoCl_2_, indicating that the synergistic effect seen is due to the composition of the glass. Surprisingly, for the glass particle conditioned media, a significantly higher amount of HIF-1α is also measured for 0Co-P + CoCl_2_ compared to the CoCl_2_ control, and for 0Co-P compared to DMEM. When comparing the ionic concentration of the conditioned media, it was found that the cobalt concentration was similar between the glass fibre and glass particle conditioned media, suggesting that the differences seen between the fibre and particle conditioned media were not due to differences in cobalt concentration. The presence of nanoparticles in the particle conditioned media may have played a role, especially as cells can internalise silicate nanoparticles (Naruphontjiralkul et al., 2018; Naruphontjirakul et al., 2019).

The effect of the silicate nanoparticles on the HIF response of keratinocytes must be fully characterised, but here they appeared to increase HIF-1α measured for both the 0Co-P and 0Co-P + CoCl_2_ conditioned media compared to the DMEM and CoCl_2_ controls, but little additional effect of these nanoparticles was seen when the glass contained Co (5Co-P). These results are in contrast to those obtained when incubating the glass fibres and particles in DMEM at a ratio of 1.5 mg ml^−1^, which showed no difference between the ionic concentrations of DMEM incubated with the glass fibres or particles. The presence of such nanoparticles has not been previously reported.

The most important result is that Co species released from the bioactive glass produces a synergistic angiogenic effect with the other glass dissolution products that is different to the Co made available when CoCl_2_ is used as the Co source in the media. The laser melting method is a promising method for producing amorphous bioactive glasses with highly degradable (low silica) compositions.

## Conclusion

Glass compositions with and without cobalt were made into fibres, with diameters from tens of nanometers to tens of microns, by laser spinning. Although the glass fibres generally behaved in a similar manner to glass particles, when incubated in DMEM for 7 days at a ratio of 1.5 mg ml^−1^, there was a clear difference in the degradation properties of the glass fibres and particles when incubated in a ratio of 6.67 mg ml^−1^. Keratinocyte cell cultures exposed to conditioned media from the fibres showed that the glasses activated the HIF pathway and promoted the expression of VEGF. Cobalt containing glass fibres caused significantly increased HIF-1α measured when compared not only to the DMEM control, but also when compared to DMEM containing the equivalent amount of cobalt chloride. The most important result was that the increase in HIF-1α was not replicated by adding cobalt chloride to conditioned media from the glass that did not originally contain cobalt. This trend was also seen in the VEGF expression, although the glass without cobalt also promoted VEGF expression compared to the DMEM control. This suggested that the Co species released from the glass produces a synergistic effect with the other glass dissolution products that is different to the Co made available when CoCl_2_ is used as the Co source in the media. The morphology of the fibres is expected to produce improved results *in vivo* as they mimic the morphology of extracellular matrix fibrils. Further characterisation of the glass particle conditioned media revealed levels of silica that are expected to be above the supersaturation point. Nanoparticles were found to form in the conditioned media in the glass particle conditioned media, but not in the fibre conditioned media, and these appeared to affect the *in vitro* cell response. Taken together, this suggests that the glass fibres developed in this study have the potential to reduce the healing time of chronic wounds, but the mechanism by which they activate the HIF pathway requires further investigation.

## Data Availability

The raw data supporting the conclusion of this article will be made available by the authors, without undue reservation. Raw data can be obtained on request from rdm-enquiries@imperial.ac.uk.
