# Supplementary material for: Cobalt containing glass fibres and their synergistic effect on the HIF-1 pathway for wound healing applications
Source: Front Bioeng Biotechnol. 2023 Mar 10;11:1125060. doi: 10.3389/fbioe.2023.1125060 (PMC10036384; doi:10.3389/fbioe.2023.1125060)
Supplement: Supplementary file 1 [file Presentation1.PDF]

Supplementary information

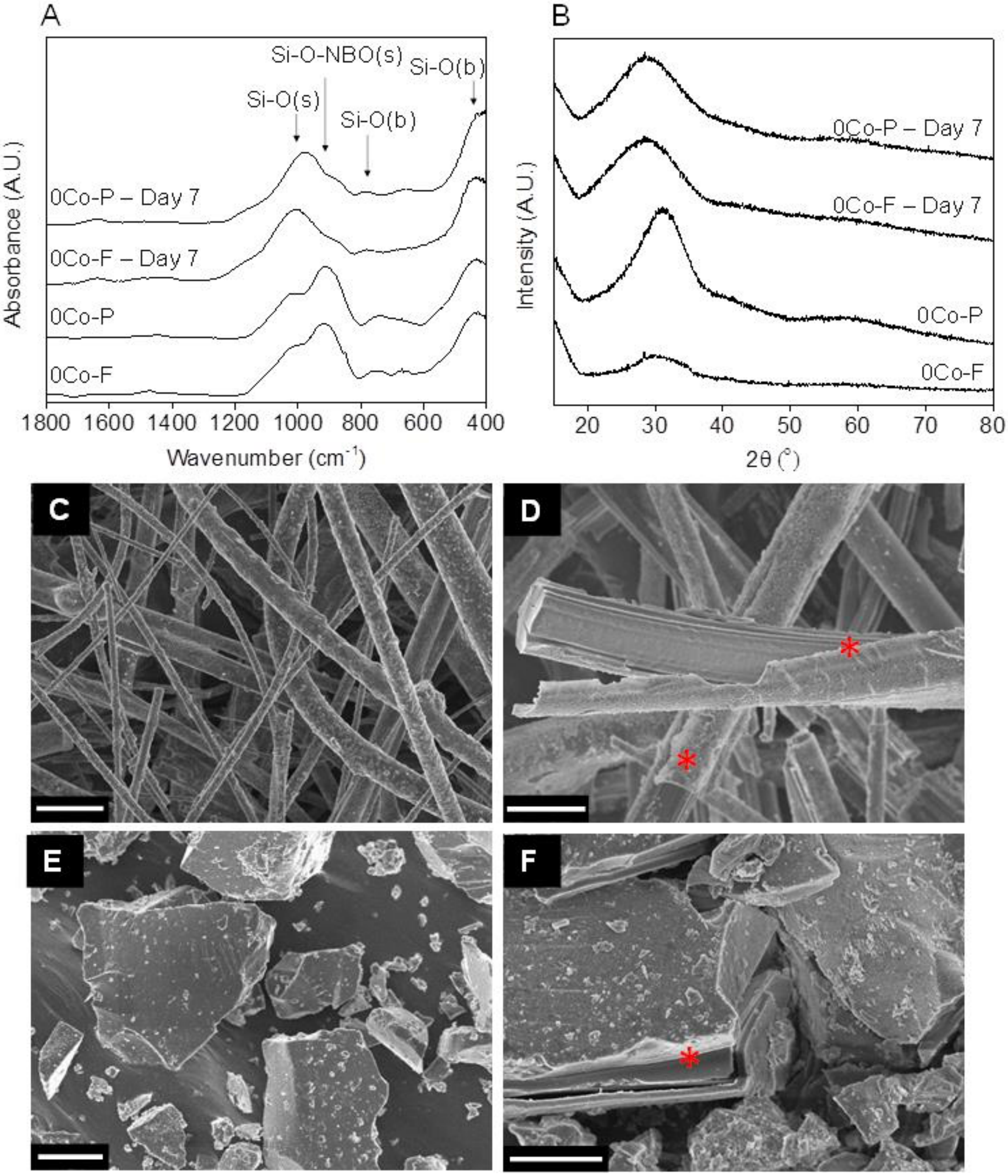

Figure S1: FTIR (A) and XRD (B) of the glasses without cobalt before and after incubation in DMEM for 7 days. 0Co fibres (0Co-F) and particles (0Co-P). SEM images of 0Co-F before (C) and after (D) incubation in DMEM, and 0Co-P before (E) and after (F) incubation in DMEM. Scale bar is 10 µm, and * indicates cracks forming in the fibres and particles after incubation in DMEM.

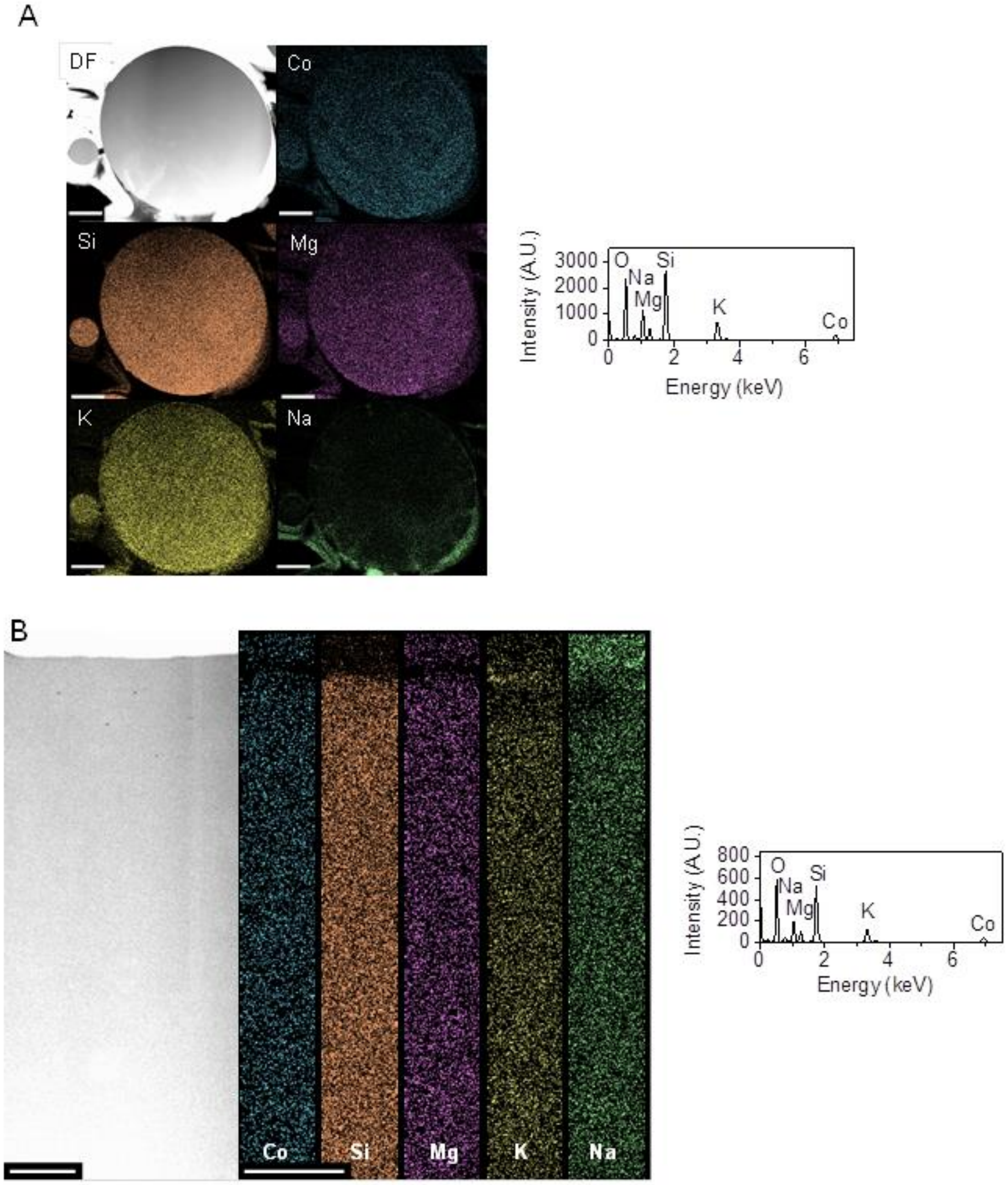

Figure S2: Darkfield TEM images and EDX spectra of the cross-sections of a 5Co-F fibre (A) and 5Co-P particle (B) before incubation in DMEM. Scale bar for (A) is 1 µm for (B) is 250 nm.

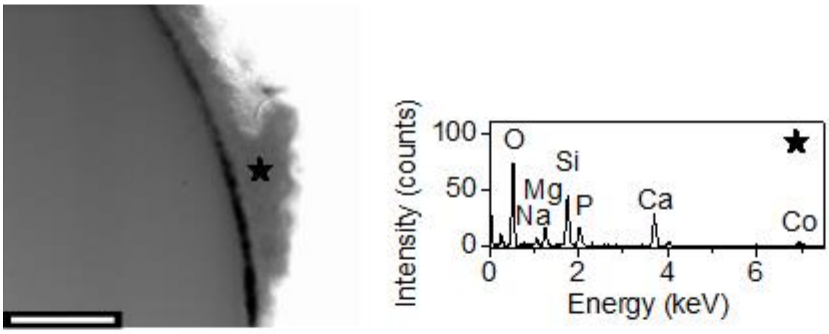

Figure S3: Darkfield TEM images of 5Co-F fiber FIB-milled cross-section and EDX spectra of the surface of 5Co glass fibre after incubation in DMEM for 7 days showing the formation of cobalt and magnesium substituted calcium phosphate layer on the fibre surface. Scale bar is 200 nm.

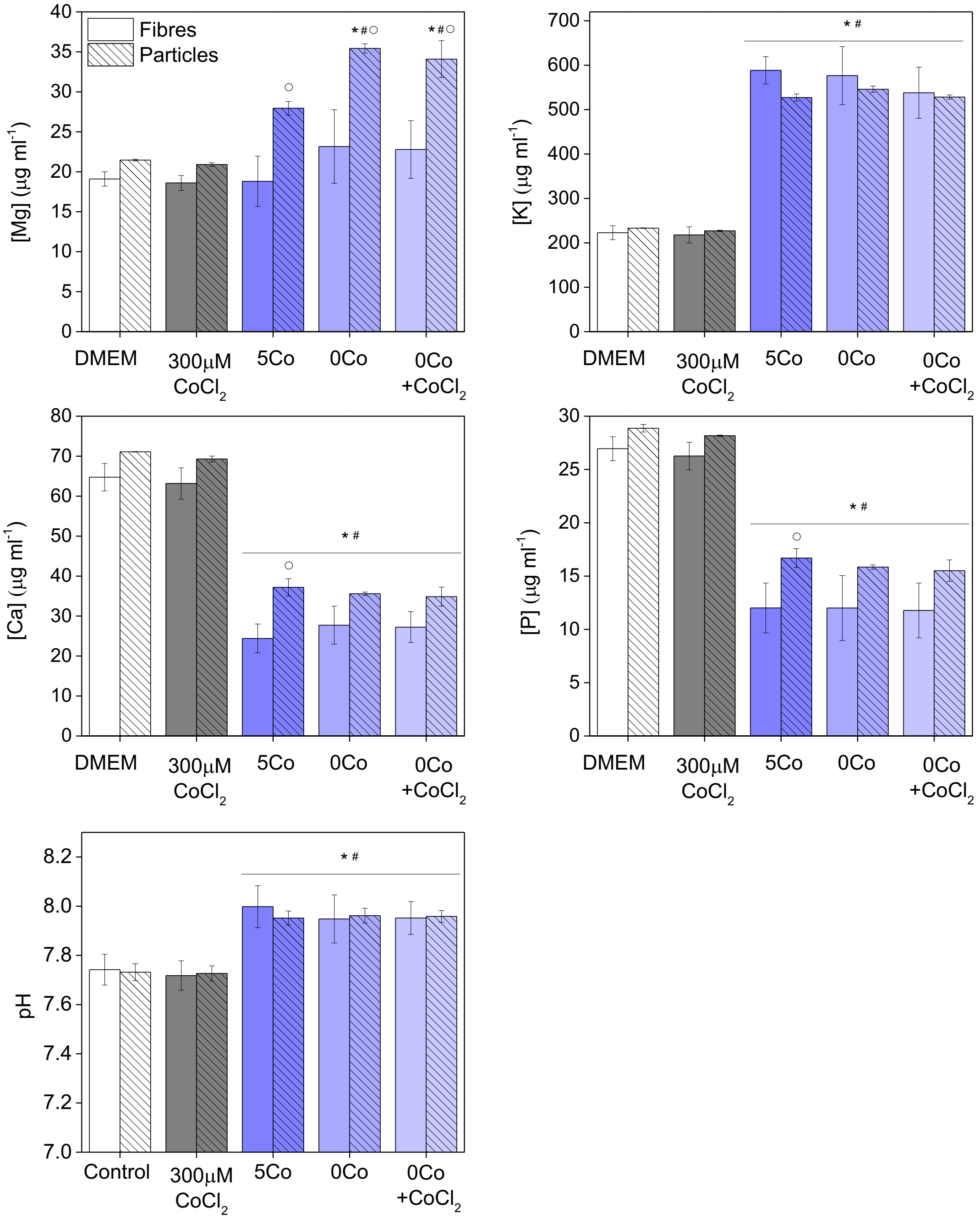

Figure S4: Ca, P, Mg and K ion concentrations and pH after equilibration overnight, of glass fibre and glass particle conditioned media after 24 hours in DMEM media, when incubating the glass in DMEM at a ratio of 6.67 mg ml^{-1}. Data are represented as mean ± standard deviation with n = 7 for the Fibre conditions and n = 3 for the Particle conditions. Statistical significance was determined with a one way ANOVA, with a Tukey means comparison test with *$p<0.05$ compared to DMEM in both the fibre and particle experiment, # $p<0.05$ compared to 300µM $CoCl_2$ controls in both the fibre and particles experiment, ○ $p<0.05$ compared to the glass particle conditioned media for the same condition in (B).
